# High Thermal Gradient in Thermo-electrochemical Cells by Insertion of a Poly(Vinylidene Fluoride) Membrane

**DOI:** 10.1038/srep29328

**Published:** 2016-07-06

**Authors:** Syed Waqar Hasan, Suhana Mohd Said, Mohd Faizul Mohd Sabri, Ahmad Shuhaimi Abu Bakar, Nur Awanis Hashim, Megat Muhammad Ikhsan Megat Hasnan, Jennifer M. Pringle, Douglas R. MacFarlane

**Affiliations:** 1 Department of Electrical Engineering, Faculty of Engineering, University of Malaya, 50603 Kuala Lumpur, Malaysia; 2 Department of Mechanical Engineering, Faculty of Engineering, University of Malaya, 50603 Kuala Lumpur, Malaysia; 3 Low Dimensional Materials Research Centre, Department of Physics, Faculty of Science, University of Malaya, 50603 Kuala Lumpur, Malaysia; 4 Department of Chemical Engineering, Faculty of Engineering, University of Malaya, 50603 Kuala Lumpur, Malaysia; 5 ARC Centre of Excellence for Electromaterials Science, Deakin University, Burwood, Victoria 3800, Australia; 6 ARC Centre of Excellence for Electromaterials Science, School of Chemistry, Monash University, Clayton, Victoria 3800, Australia

## Abstract

Thermo-Electrochemical cells (Thermocells/TECs) transform thermal energy into electricity by means of electrochemical potential disequilibrium between electrodes induced by a temperature gradient (ΔT). Heat conduction across the terminals of the cell is one of the primary reasons for device inefficiency. Herein, we embed Poly(Vinylidene Fluoride) (PVDF) membrane in thermocells to mitigate the heat transfer effects - we refer to these membrane-thermocells as MTECs. At a ΔT of 12 K, an improvement in the open circuit voltage (V_oc_) of the TEC from 1.3 mV to 2.8 mV is obtained by employment of the membrane. The PVDF membrane is employed at three different locations between the electrodes i.e. x = 2 mm, 5 mm, and 8 mm where ‘x’ defines the distance between the cathode and PVDF membrane. We found that the membrane position at x = 5 mm achieves the closest internal ∆T (i.e. 8.8 K) to the externally applied ΔT of 10 K and corresponding power density is 254 nWcm^−2^; 78% higher than the conventional TEC. Finally, a thermal resistivity model based on infrared thermography explains mass and heat transfer within the thermocells.

Thermoelectricity, a phenomenon where a temperature gradient is converted into electricity, is a topic of intense research interest primarily for energy harvesting applications. A conventional thermoelectric (TE) module consists of an array of p- and n- type semiconducting materials assembled between two electrodes maintained at different temperatures. The efficiency of a typical TE device is governed by the temperatures of the hot and cold electrodes (Th and Tc) as well as the intrinsic properties of TE materials. Thus, a dimensionless figure of merit (ZT) is defined by equation (1) as a quantitative measure of energy conversion capability of TE materials (p- and n- type materials).


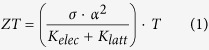


where “σ” is electrical conductivity (S/m), “α” is the thermoelectric, or “Seebeck”, coefficient (V/K), “T” is the absolute temperature (K) while K_elec_ and K_latt_ are the electronic and lattice contribution of thermal conductivity (W/m·K) of the material, respectively^1^,^2^,^3^,^4^. Therefore, the overall device efficiency (*η*) in the literature^3^,^4^ is expressed as:


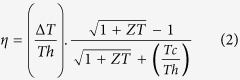


Researchers have subjected intensive efforts to attain higher ZT values through various routes^1^,^2^,^5^,^6^,^7^,^8^,^9^ but the highest value in bulk materials is around 1.4–1.6^6^,^7^ which is still insufficient for large scale industrial applications. The foremost challenge to exceed the ZT value is to overcome the interdependency of electronic and thermal properties of the materials. Equation (1) suggests that a high ZT is dependent on high electrical conductivity (σ) and Seebeck coefficient (α), and low thermal conductivity (K). Furthermore, an increase in charge carrier concentration (n, cm^−3^) will have a proportional effect of increasing σ, but decreasing α. Thus, there is a significant tradeoff between the three key parameters σ, α and K, where typically a material of high electrical conductivity will also have high thermal conductivity, but consequently low ZT. Strategies such as phonon glass electron crystal (PGEC) structures, quantum dots and superlattices have been devised in order to provide both high electrical conductivity and low thermal conductivity^1^,^2^,^5^,^6^,^7^,^8^,^9^.

In recent times, novel thermoelectric effects apart from the semiconductor-based Seebeck effect have been introduced, such as the Spin Seebeck effect^10^ and the electrochemical Seebeck effect. The electrochemical Seebeck effect arises in thermo-electrochemical cells, TECs, when a solution containing a redox couple is subjected to a temperature gradient. The temperature gradient between the electrodes disturbs the electrochemical potential equilibrium between the electrolyte and electrode surface causing the current to flow when the circuit is complete^11^. This effect can result in significantly high Seebeck coefficients of redox electrolytes, which translate into potentially high power outputs. Furthermore, these solutions generally possess low thermal conductivity, which can contribute to a higher thermoelectric figure of merit.

TECs, traditionally known as thermo-galvanic cells, have been studied since the early 1980s^12^,^13^,^14^. TECs generate steady electric current by the virtue of a temperature gradient between the two electrodes placed in an electrolyte. Conventionally, characterization of the electrochemical Seebeck coefficient, S_e_, is conducted using a two-compartment setup, connected by a salt bridge, which allows a physical pathway for ion transfer between the two compartments. The two compartments are subjected to a temperature gradient (ΔT), and ion migration is enabled from the hotter compartment to the colder compartment through the salt bridge (Fig. 1(a)). The resulting potential difference (ΔV) is used as the basis for the calculation of S_e_ (where S_e_ = ΔV/ΔT)^11^,^15^.

For application of these devices, the TEC is used as a cell structure as shown in Fig. 1(b) where the electrolyte and both the electrodes are confined in a single chamber. The ion transport within the cell configuration is a complex combination of natural convection, migration, thermal and chemical diffusion. Although, cell configuration is practically more applicable but maintaining the temperature gradient between electrodes is a key challenge which limits the performance of the TEC, as the thermal conductivity of the electrolyte solution will inevitably reduce ΔT. On the contrary, the two-beaker setup (Fig. 1(a)) allows maintaining high temperature gradient as the two compartments are separated and can be easily maintained at different temperatures. Typical strategies to maintain the integrity of the temperature gradient in the “cell configuration” include an auxiliary cooling system on the cold side electrode, which adds complexity, cost and bulk to the overall system. In this work, we insert and optimize the position of a PVDF membrane within the TEC to maintain the temperature gradient without any need for an auxiliary cooling system. Hu *et al*.^16^ have utilized commercial glass-frits and NOMEX HT4848 (a nylon-based polymer separator commercially prepared by NOMEX, DuPont) in their thermocells as separators between their anode and cathode. They attributed the obtained high output power density of 1.8 W/m^2^, and corresponding Carnot efficiency of 1.4%, to high electron transfer rates between their novel carbon nanotube electrodes and the electrolyte. Thus, the influence of the different separators on thermal gradient and electrochemical performance were not been studied in depth.

An ideal separator should be porous in order to allow ionic transport, yet highly thermal resistive in order to maintain a large temperature gradient between the electrodes. PVDF was chosen for this study as it is an excellent thermal insulator and is also highly chemically stable polymer, in particular with respect to the redox couple. Additionally, films of PVDF can be easily fabricated through facile methods such as phase inversion and electrospinning. PVDF has also been used in energy storage and conversion applications such as batteries and fuel cells^17^,^18^,^19^. For example, separators of PVDF-co-hexafluoropropylene (PVDF-HFP), sandwiched poly(m-phenylene isophthalamide) (PMIA) between two PVDF layers (PVDF/PMIA/PVDF) and sandwiched PVDF layer between two poly(phthalazinone ether sulfone ketone) (PPESK) (PPESK/PVDF/PPESK) have been studied for Li-ion batteries^17^,^18^,^19^. Researchers have also explored PVDF membranes in fuel cells and piezoelectric devices^20^,^21^. However, to the best of our knowledge, PVDF membranes have not been studied for TEC applications.

## Results

**Figure 1:**
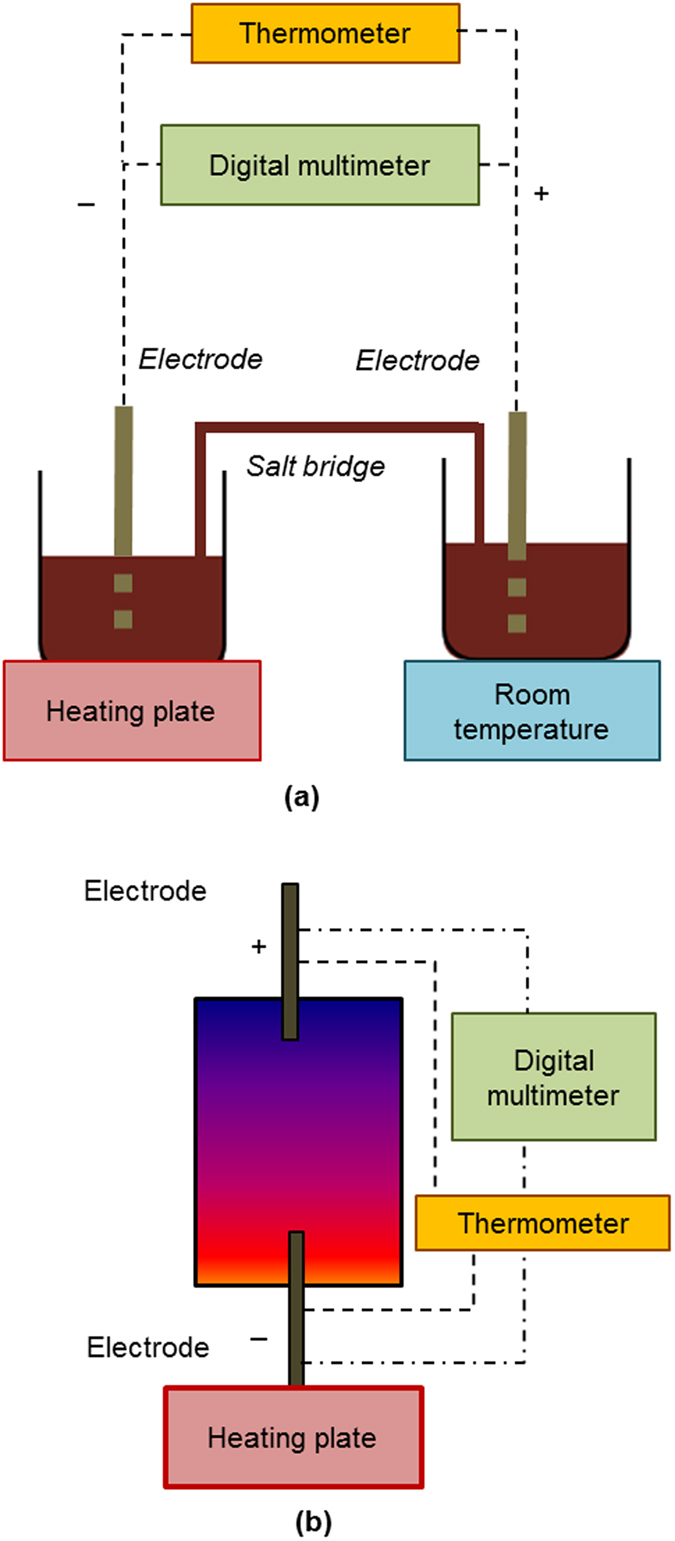
(**a,b**) Schematic illustration defining the working principle of thermo-electrochemical cells. In (**a**) a two compartment arrangement is shown where two electrodes are placed in two separate compartments with the same electrolyte. A temperature gradient between the two electrodes is created by heating one of the compartments while cooling or maintaining the other at room temperature. A salt bridge, containing the same electrolyte, is placed between the hot and cold compartment to allow movement of the redox species and counter-ions between the two electrodes. (**b**) Shows the TEC device design that is more realistic for practical applications. Rather than having separate compartments, the two electrodes and the electrolyte are assembled in a single cell. The orientation of the electrodes (either vertical or horizontal) and the heating scheme (heating the bottom electrode or the upper) can be varied and literature for all techniques is available. However, comparison between the heating schemes shows that heating from the bottom always produces better results. In both of the thermocell designs (**a**,**b**) the electrodes are connected with a digital multimeter to measure the open circuit voltage. The temperatures of the respective electrodes are measured through digital thermometers.

**Figure 2:**
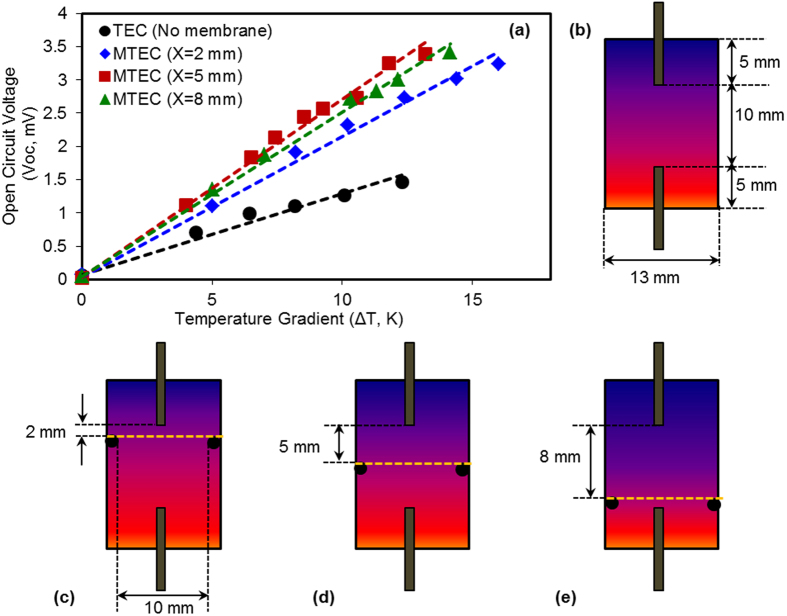
(**a–e**) Thermo-electrochemical performance of TECs and MTECs. The cell configuration thermocell (as shown in Fig. 1(b)) was assembled with two Graphite electrodes and I^−^/I_3_^−^ aqueous redox solution as the electrolyte. The open circuit voltage (V_oc_) was measured once the temperature gradient became stable. The temperature of bottom electrode was gradually increased while maintaining the upper electrode at room temperature. The best performance was observed when the membrane was held midway between the two electrodes (x = 5 mm, shown by the red line on the plot). The TEC and all MTECs exhibit a linear relation between the temperature gradient and the open circuit voltage. (**b**) Design specifications of TEC (when there is no membrane between the hot and cold electrode. (**c–e**) Design specifications of MTEC (when membrane is placed between the hot and cold electrode). The effect of membrane position is studied in this work. (**c**) When the membrane is closer to the cold electrode (i.e. x = 2 mm) (**d**) when membrane is in between the two electrodes (i.e. x = 5 mm) and (**e**) when membrane is closer to the hot electrode (i.e. x = 8 mm). In all the TEC and MTEC cases the distance between the electrodes and electrode surface area were kept constant to be 10 mm and 0.34 cm_2_, respectively.

**Figure 3:**
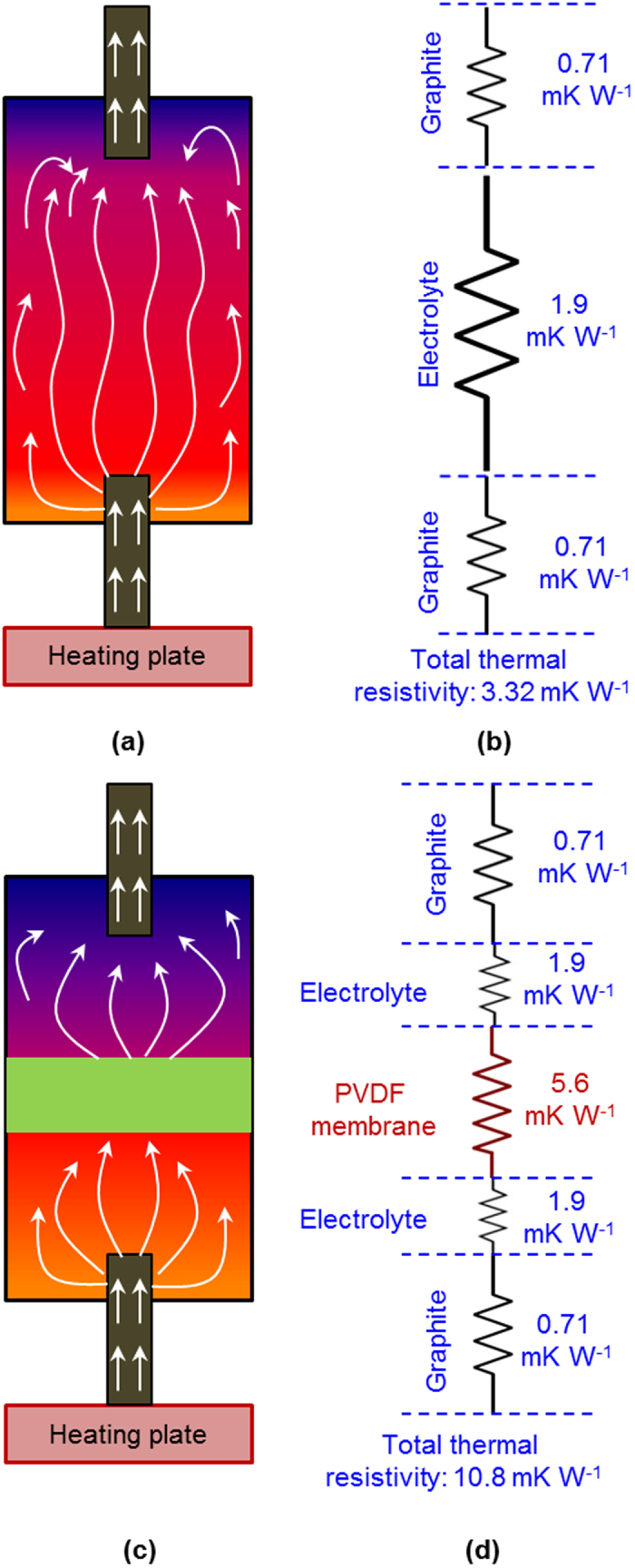
(**a–d**) Illustration of heat propagation and thermal resistivity models for TEC and MTEC cases. (**a**) Shows the heat flow, represented by white arrows, in the form of natural convection. In (**b**) the different contribution to the total thermal resistivity of the TEC is estimated. The total thermal resistivity is 3.32 m.K.W^−1^. (**c**) Illustrates the heat flow in the MTEC when the membrane is placed equidistant between the two electrodes. The role of the PVDF membrane in the MTEC is to hinder the heat dissipation between the electrodes, keeping the electrodes closer to the actually applied external temperature values. The thermal resistivity model of the MTEC shown in (**d**) illustrates that owing to higher thermal resistivity of PVDF membrane i.e. 5.6 m.K.W^−1^, the overall thermal resistivity is increased to 10.8 m.K.W^−1^, decreasing the heat flow.

The electrochemical Seebeck coefficient (S_e_) for the 0.7 M I^−^/I_3_^−^_(aq)_ redox couple was 0.4 mV/K when tested in the two compartment cell (Fig. 1(a)). This is 0.13 mV/K less than the reported value of 0.53 ± 0.04 mV/K for a 0.4 M solution, consistent with the expectation that for this range of concentrations, an increase in molarity results in a decrease in S_e_^15^,^22^,^23^. However, highly concentrated solutions are preferred for higher power outputs from the thermocells^15^,^24^,^25^. The designs of thermocells used in present work and the thermo-electrochemical performances of TECs (membrane less) and MTECs (membrane inserted TEC) are depicted in Fig. 2. The maximum open circuit voltage (V_oc_) generated by the conventional TEC is 1.3 mV at the temperature gradient of 12 K. However, the introduction of a PVDF membrane in the TEC significantly enhances the V_oc_ to 2.7 mV for the case of x = 5 mm, at the same value of externally applied ΔT (Fig. 2(a)). The details of open circuit voltages from the TEC and MTECs are given in Table 1. The origin of this higher performance is enhancement of the thermal gradient between the two electrodes due to the presence of the PVDF membrane, which has the effect of decreasing convective flow between the electrodes. By creating a thermal barrier between the two halves of the cell the temperature at the working surfaces of the electrodes are able to more closely approach the externally applied temperatures, thus maximizing the internal thermal gradient and achieving the maximum V_oc_. An illustration of the heat flow in a TEC and MTEC are presented in Fig. 3(a,c) and the thermal barrier is quantitatively expressed as a thermal resistivity (Fig. 3(b,d)).

In this paper, we first discuss the influence of PVDF membrane on the power generation characteristics of the TEC and MTECs. Then, visualisation of the mass and heat transport within the TEC and MTEC using infrared thermography allows insight into the role of the membrane separator in these cells.

### Power Generation characteristics of TEC and MTEC

**Figure 4:**
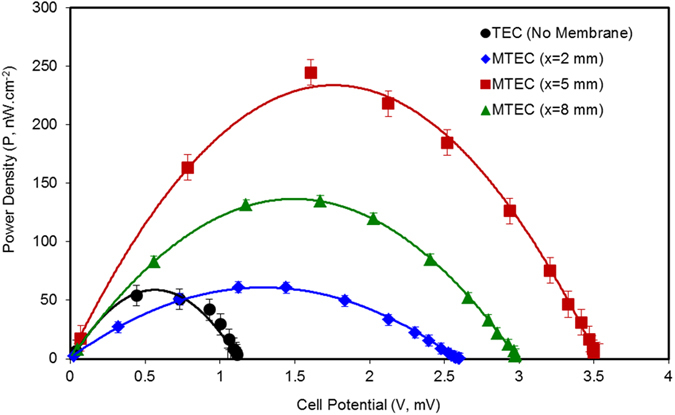
Power curves for TEC and MTECs. TEC and MTECs were maintained at a temperature gradient of 10 K and connected to an external load. Individual resistances (R, Ω) were applied and the corresponding cell potentials were recorded. Power outputs were calculated using Ohm’s law (P = V^2^/R). The black line shows the performance of the TEC, where the maximum power density was 54 nW/cm^2^. It was found that employment of the PVDF membrane significantly enhances the power output performance. Although the maximum power density is only slightly increased (i.e. 61 nW/cm^2^) when membrane is at x = 2 mm (i.e. closer to cold electrode), the cell open circuit potential is clearly improved, from 1.1 mV to 2.6 mV. Furthermore, the power generation from MTECs is related to the position of the membrane. The highest performance in terms of maximum power output and cell potential is observed when the membrane is held midway between the electrodes (i.e. x = 5 mm). The maximum power density in this case was 245 nW/cm^2^ and the cell potential was 3.5 mV. At x = 8 mm, when the membrane was placed closer to the hot electrode, the performance was reduced compared to the x = 5 mm case, but was still better than the TEC or x = 2 mm case. The power performance of MTECs is strongly dependent on the temperature gradient achieved in each case. The maximum temperature gradient is achieved at x = 5 mm and correspondingly the power density is the highest. Similarly, MTEC with x = 2 mm produces a lesser temperature gradient and thus has the lower performance.

Inserting the PVDF membrane into the TEC has the direct benefit of enhancing the temperature gradient, and hence the generated voltage, as illustrated in Fig. 2(a). These results are summarised in Table 1, where an improvement of 52% was achieved for the case of membrane at x = 5 mm compared to the TEC. Consequently, the overall thermo-electrochemical power generation of the MTEC is shown to be enhanced significantly compared to the TEC as expressed in Fig. 4. The maximum power density from the MTEC was 245 nWcm^−2^, achieved for the case of x = 5 mm, measured at temperature gradient of 10 K, which is almost four times higher than the TEC maximum power density (54 nWcm^−2^).

It is to be noted that each membrane configuration in MTECs results in improved cell potential as compared to the TEC. In Table 1, the calculated thermal gradient between the electrodes is presented, using ΔT_calc_ = ΔV/S_e_, where S_e_ = 0.4 mV/K and ΔV in each case is the open-circuit voltage recorded for the cases of x = 2 mm, 5 mm, and 8 mm respectively. Table 1 shows that a TEC is only able to provide a temperature gradient of 2.7 K between the electrodes, whereas the externally applied temperature gradient is 10 K. This observation further confirms the rapid heat flow in the TEC cases. In all the MTECs the thermal gradient between the electrodes is improved as compared to the TEC, reaching an optimal temperature gradient (8.8 K) for x = 5 mm. Consequently, this MTEC provides the best power performance. Additionally, the lowest power generation of 61 nWcm^−2^ was achieved with the x = 2 mm MTEC, when the membrane is closer to the cold electrode, which has the smallest thermal gradient amongst the membrane inserted cases.

### Mass and heat transfer characteristics of the TEC

**Figure 5:**
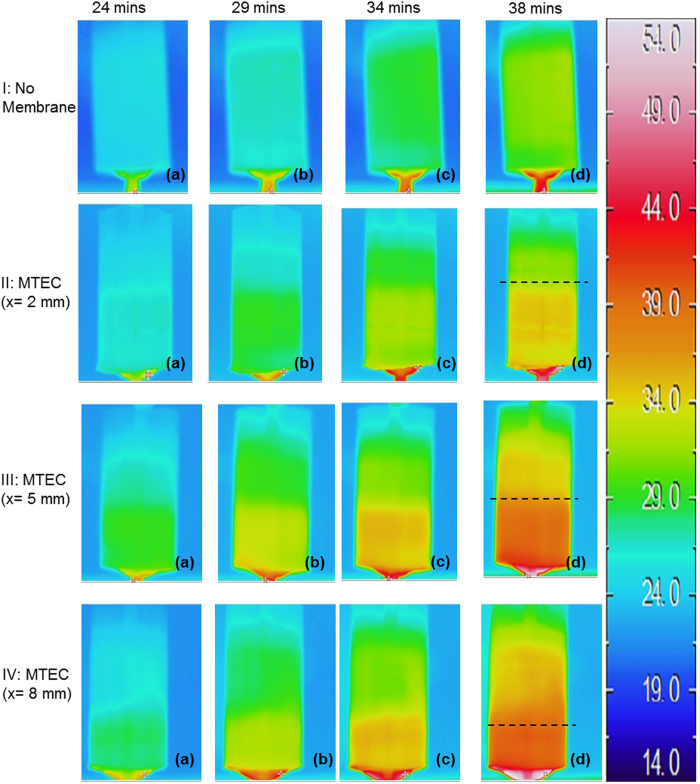
Infrared thermography of TEC and MTECs. The series I, II, III, and IV represents no membrane, membrane closer to cold electrode, membrane at the center between the electrodes, and membrane closer to hot electrode respectively. Thermal images of each TEC and MTEC were taken after 24, 29, 34 and 38 minutes of heating. During the experiments the heating rate of the cells and ambient conditions were kept identical for all cases. The thermal images are consistent with the heat flow and thermal resistivity models in Fig. 3. In series I (i.e. no membrane), the temperature of the electrolyte overall increases but without maintaining any temperature gradient. However, in series II, III and IV the PVDF membrane creates distinct temperature zones with different temperatures, which ultimately improves the performance. The highest temperature gradient is created in series III when the membrane is place equidistant from the two electrodes thus it also has the highest electrochemical performance.

**Figure 6:**
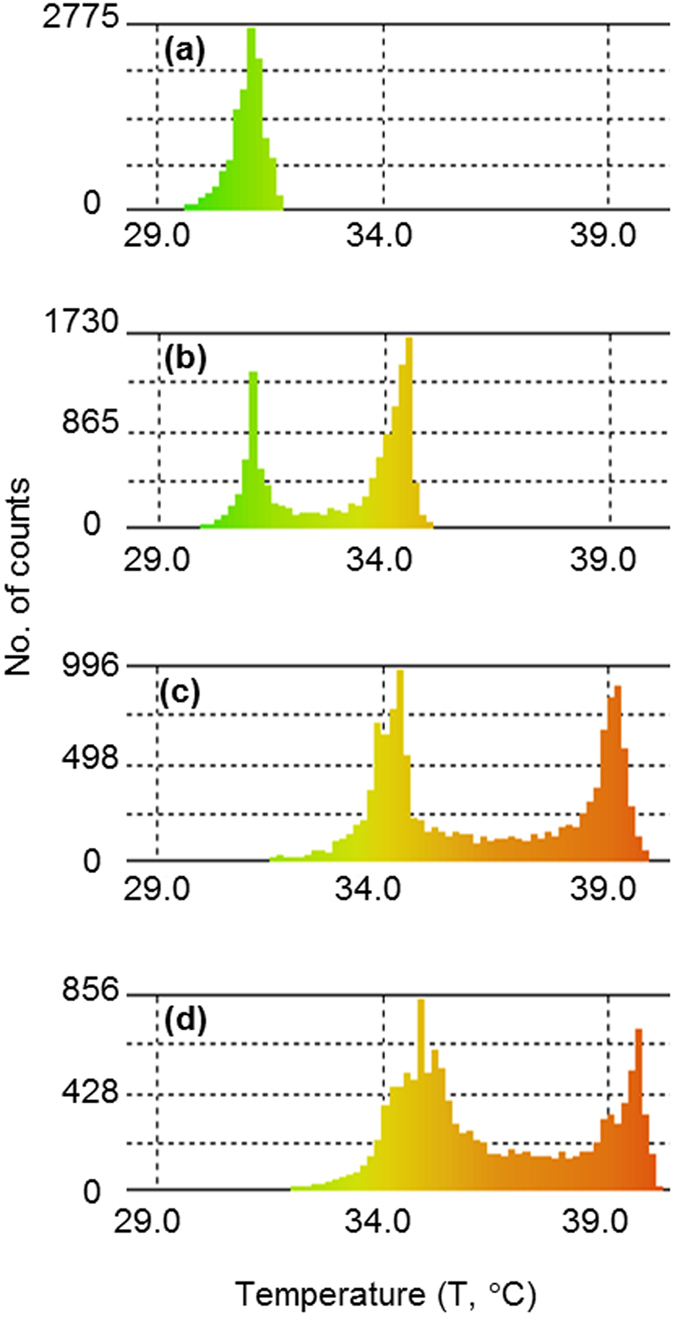
(**a–d**) Temperature histograms of the TEC and MTECs. The thermal imaging software InfRec Analyzer NS9500, was used to produce histograms for each TEC and MTECs maintained with the cold and hot electrode at 30 and 40 °C, respectively.

Reduction, in the case of the (I^−^/I_3_^−^) redox couple, occurs at the colder electrode (i.e. the cathode). The reduced species, migrate towards the anode where they oxidize; releasing the electrons^13^,^14^. This can be visualised in the infrared thermal images of the TECs in Fig. 5(I), where the convection effects coupled with the thermal diffusion allows fast equilibration of the temperature within the cell. After 29 mins of heating the average temperature of the redox solution was 26.7 °C (±1 °C). Thus, in the case of the TEC the heat transfer through mass (fluid) flow rapidly diminishes the temperature gradient across the TEC that is critical to the its operation. The images of Fig. 5(I) indicated that after 34 and 38 mins the temperature of the solution has been increased to 28.4 °C (±1 °C) and 31.0 °C (±1 °C), and thus the temperature gradient across the vertical axis has been significantly reduced. The maximum temperature difference was achieved after 38 mins of heating, thus resulting in the maximum V_oc_. Figure 6 shows a quantitative representation of temperature distribution within the cell, extrapolated from Fig. 5 using thermal imaging software after 38 mins of heating. In the histogram of the TEC, the temperature of the electrolyte is concentrated around ca. 30.4–31.8  °C (±1 °C) resulting in a unimodal distribution (Fig. 6(a)). The unimodal temperature distribution supports the weaker temperature gradient maintained between the electrodes of TEC.

### Mass and heat transfer characteristics of the MTECs

The effect of the insertion of a PVDF membrane on the heat flow is illustrated in Fig. 5(II,III,IV) for the cases of membrane position 2 mm, 5 mm, and 8 mm, respectively. The PVDF material is an efficient thermal insulator with thermal resistivity of ~6 mK W^−1^ 26,27,28. The thermal resistive effects of PVDF in MTECs obstruct the heat flow between the electrodes creating distinct temperature zones, thus resulting in bimodal temperature distribution in MTECs, as shown in Fig. 6(b,c,d). In the TEC after heating for 38 mins the average temperature of the electrolyte was 31.0 °C (±1 °C), whereas after the same heating time in the MTECs temperature zones of 31 °C and 35 °C (±1 °C) in the x = 2 mm case, 34 °C and 39 °C (±1 °C) in the x = 5 mm case, and 35 °C and 40 °C (±1 °C) in the x = 8 mm case were observed (Fig. 6(b,c,d)). The details of the peak positions and breadths are given in Table 1. Therefore, in summary, the presence of the membrane acts as a thermal resistor to hinder heat flow. However, the improved open circuit voltage and maximum power density also indicate that ion transfer between the electrodes is not adversely affected by the PVDF barrier.

### Thermal resistance model

To further elucidate the effect of the PVDF membrane inclusion in the TEC, a thermal resistance model is proposed in Fig. 3(b,d). A temperature gradient is key to the voltage generation in a TEC, and in an ideal case this temperature gradient is maintained throughout the experiment and is equivalent to the difference in applied temperatures at the electrodes. However, more realistically, losses occur due to a number of factors: temperature gradients in the electrodes, heat lost to the environment, and convection and conduction within the solution. For TEC the equivalent thermal resistance circuit can be represented by Fig. 3(b). For the solution, the thermal resistance may be described in terms of its thermal conductivity and thermal mass transfer (predominantly convection). In this case, the relatively high convection of the solution can be represented by a low thermal resistance. As noted previously, it results in the solution reaching thermal equilibrium relatively quickly within the TEC, which significantly diminishes the temperature gradient and the power output to 2.7 K and 54 nWcm^−2^, respectively. In comparison, the equivalent thermal resistance of the MTEC is represented in Fig. 3(d), where the thermal resistance of the PVDF membrane is much larger than those of the solution or the electrodes. Furthermore, the presence of the membrane also has a direct impact on the thermal resistance of the solution: whilst the thermal conductivity may be assumed as constant, the thermal mass transfer effects (i.e. convection) is diminished as the volume is now physically divided into two half cells. Consequently, the thermal resistance of the solution is also increased prompting MTEC closer to the applied temperature gradient i.e. 8.8 K. Of further note is that the optimum performance of the cell (245 nWcm^−2^) is achieved at a membrane position of x = 5 mm, i.e. equidistant from both electrodes, where the power output in this configuration of membrane has provided 78% improvement to the performance of the TEC. This observation can also be explained within the context of limiting the convection within the half cells, as this configuration allows the minimum electrolyte volume on either side of the membrane.

## Discussion

We have demonstrated here that a separator membrane significantly affected the operation of a thermo-electrochemical cell, by reducing the effect of convective currents in the electrolyte. A thermal resistance model shows that the dominant thermal resistance of the membrane reduces temperature drops in other parts of the cell, i.e. across the two electrolyte layers and across the electrode assembly. The findings of this work suggest that the insertion of the membrane at its optimum configuration is able to improve the temperature gradient by 70% and the resulting power density by 78%. These results will be useful to optimize the future performance of TECs when used in conjunction with the optimised formulations of redox couples for waste heat conversion into electricity.

## Methods

### Materials

**Figure 7:**
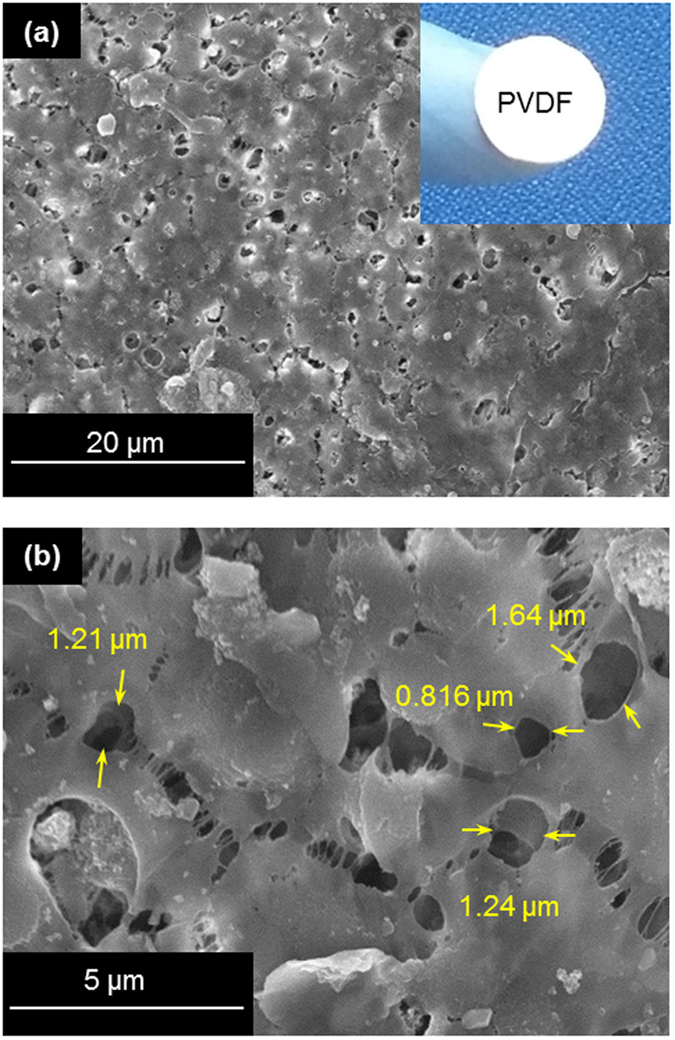
(**a,b**) FE-SEM micrographs of PVDF membrane. (**a**) Low magnification. (**b**) High magnification shows the pore size ranges from 0.8 to 1.6 μm. The inset of (**a**) is the snapshot of the PVDF membrane used.

The iodide/triiodide (I^−^/I_3_^−^) redox solution (0.7 M) was prepared in 50 ml distilled water by dissolution of Iodine (5 g, Sigma Aldrich; ≥99.8%) and Potassium Iodide (10 g, Sigma Aldrich; ≥99.5%). The solution was heated to 50 °C for 10 mins to ensure proper solubility. A 200 μm thick PVDF membrane was prepared by phase inversion method. Commercially available Poly(Vinylidene Fluoride) (PVDF, Kynar^®^ K-761, molecular weight of 440,000, density = 1.7 g/ml and melt temperature ~165 °C) powder was mixed with 1-Methyl-2-Pyrrolidinone (NMP) in a ratio of 18% by wt. The solution was stirred for 24 hours at 60 °C. The polymer solution was then cast on a glass plate and the resultant flat sheet membranes were immediately immersed in a coagulation bath containing deionised water. The prepared PVDF membrane is porous with pore size ranging in several microns as shown in Fig. 7.

### Measurement of the Seebeck coefficient of the I^−^/I_3_^−^ aqueous redox solution

The prepared I^−^/I_3_^−^ aqueous redox solution was put in two compartments (Pyrex glass beakers), according to the schematic shown in Fig. 1(a). Each compartment contained a Pt electrode. The physical exchange of the solutions between the two compartments was established by providing a salt bridge containing the same solution. Pt electrodes were cleaned with concentrated HNO_3_ followed by methanol rinsing. The thermal gradient was created by heating one of the compartments through an electrically operated heater at 0.2 °C/min rate while maintaining the other at room temperature (Fig. 1(a)).

### Morphological and Heat Transfer Characterization

The surface morphology of PVDF membrane was studied by FE-SEM (HITACHI SU8030). Infrared Thermography was carried out at the framerate of 8.5 Hz through R300SR-HB (Nippon Avionics Co. Ltd.) maintaining similar environmental conditions to comprehend the role of membranes in enhancing thermal gradient within the thermocells.

### Thermo-Electrochemical Cell Fabrication and Measurements

Thermo-electrochemical performance of the redox solution with PVDF membranes was studied in a 13 mm diameter and 20 mm length cells (Fig. 2(b)). The two open ends of the thermocell were closed by rubber sealants to ensure no leakage and the redox solution was inserted through a syringe. The distance between the vertically positioned graphite electrodes were kept constant at 10 mm while the membrane positions (x) were altered between 2 mm, 5 mm and 8 mm away from the top (cold) electrode. For MTECs, a rubber O-ring (ϕ_out_ = 13 mm and ϕ_in_ = 10 mm) was used to hold the membrane in the intended positions (Fig. 2(c,d,e)). The reproducibility of the results was examined and the difference in Seebeck values, on average, was found to be ±0.02 mV/K. The electrodes were cleaned by abrasive paper followed by ultrasonic cleaning with acetone. The thermocells were heated in cold-above-hot arrangement at 0.2–0.5 °C/min and the open circuit voltage (V_oc_) was measured by an Agilent 34461A 6_1/2_ digital multimeter. The electrochemical Seebeck coefficient was evaluated by the slope of the linear fit between V_oc_ and ΔT. For power output measurements the TEC and MTECs were connected to a variable resistor box (Elenco RS-500), different resistive loads (R, Ω) were applied and the corresponding cell potentials (V, mV) were recorded. The Ohm’s law (P = V^2^/R) was used to calculate the power values.

## Additional Information

**How to cite this article**: Hasan, S. W. *et al*. High Thermal Gradient in Thermo-electrochemical Cells by Insertion of a Poly(Vinylidene Fluoride) Membrane. *Sci. Rep.* **6**, 29328; doi: 10.1038/srep29328 (2016).

## Figures and Tables

**Table 1:** Summary of the TEC and MTEC results.

		(a) Data from Fig. 2a (Electrochemical measurements)				(c) Data from Fig. 6 (Infra-red thermal images)	
								(b) Data from Fig. 4 (Power Curves)			Peak 1	Peak 2
		Results summary		Open Circuit Voltage (V_oc_, mV) *@ 12 *K)	Max. Cell Potential (V, mV)						Max. Power Output (nW/cm^2^)	Calc. Thermal gradient (ΔT_calc_, K)	Range ( °C)	Range ( °C)
TEC	No Membrane	1.3	1.1	54	2.7	30.4–31.8	–
MTEC	X = 2 mm	2.6	2.6	61	6.5	30.9–31.5	34.0–34.8
	X = 5 mm	2.7	3.5	245	8.8	33.7–34.8	38.7–39.6
	X = 8 mm	2.8	3.0	135	7.5	34.0–35.6	39.0–40.1

The S_e_ of the electrolyte was 0.4 mV/K. (a) The values of open circuit voltages are summarized from Fig. 2(a) when T_hot_ = 37 °C and T_cold_ = 25 °C. (b) Data extracted from Fig. 4: Maximum cell potential and output power density. Calculated temperature gradient between the electrodes under load conditions, where ΔT_calc_ = V/S_e_. (c) Peak details of temperature histograms as shown in Fig. 6. For (b) and (c) all TEC and MTEC were maintained with T_hot_ = 40 °C and T_cold_ = 30 °C.

## References

[b1] Synder G. J. & Toberer E.S. Complex thermoelectric materials. Nature Materials 7, 105–114 (2008). 18219332 10.1038/nmat2090

[b2] Tritt T. M. Thermoelectric phenomena, materials and applications. Annu. Rev. Mater. Res. 41, 433–448 (2011).

[b3] Telkes M. Efficiency of thermoelectric generator. Jour. Appl. Phys. 18, 1116–1127 (1947).

[b4] Chasmar R. P. & Stratton R. Thermoelectric figure of merit and its relation to thermoelectric generator. Jour. Elec. Contr. 7, 52–57 (1959).

[b5] Gonzalez M. M-., Calero O. C-. & Chao P. D-. Nanoengineering thermoelectrics for 21st century: Energy harvesting and other trends in the field. Renewable and Sustainable Energy Reviews 24, 288–305 (2013).

[b6] Poudel B. . High-thermoelectric performance of nanostructured Bismuth Antimony Telluride bulk alloys. Science 320, 634–638 (2008). 18356488 10.1126/science.1156446

[b7] Xie W. . High performance Bi_2_Te_3_ nanocomposites prepared by single-element-melt-spinning spark-plasma sintering. J. Mater. Sci. 48, 2745–2760 (2013).

[b8] Hicks L. D. & Dresselhaus M. S. Thermoelectric figure of merit of a one-dimensional conductor. Phys. Rev. B. 47, 16631–16634 (1993). 10.1103/physrevb.47.16631 10006109

[b9] Venkatasubramanian R., Siivola E., Colpitts T. & O’Quinn B. Thin-film thermoelectric devices with high room-temperature figures of merit. Nature 413, 597–602 (2001). 11595940 10.1038/35098012

[b10] Uchida K. . Observation of the spin Seebeck effect. Nature 455, 778–781 (2008). 18843364 10.1038/nature07321

[b11] Gunawan A. . Liquid thermoelectrics: Review of recent and limited new data of thermogalvanic cell experiments. Nanoscale and Microscale Thermophysical Engineering 17, 304–323 (2014).

[b12] Chum H. L. & Osteryoung R. A. Review of thermally regenerative electrochemical systems. Technical report. Available at: http://www.nrel.gov/docs/legosti/old/416_v1.pdf. (Accessed: 15th November, 2015) (1981).

[b13] Quickenden T. I. & Mua Y. The power conversion efficiencies of a thermogalvanic cell operated in three different orientations. J. Electrochem. Soc. 142, 3652–3659 (1995).

[b14] Mua Y. & Quickenden T. I. Power conversion efficiency, electrode separation, and overpotential in the Ferricyanide/Ferrocyanide thermogalvanic cell. J. Electrochem. Soc. 143, 2558–2564 (1996).

[b15] Abraham T. J., MacFarlane D. R. & Pringle J. M. Seebeck coefficients in ionic liquids–prospects for thermo-electrochemical cells”. Chem. Commun. 47, 6260–6262 (2011). 10.1039/c1cc11501d 21544302

[b16] Hu R. . Harvesting waste thermal energy using a Carbon-Nanotube-Based thermo-electrochemical cell. Nano Lett. 10, 838–846 (2010). 20170193 10.1021/nl903267n

[b17] Pu W., He X., Wang L., Jiang C. & Wan C. Preparation of PVDF-HFP microporous membrane for Li-ion batteries by phase inversion. J. Membr. Sci. 272, 11–14 (2006).

[b18] Zhai Y. . Sandwich-structured PVdF/PMIA/PVdF nanofibrous separators with robust mechanical strength and thermal stability for lithium ion batteries. J. Mater. Chem. A 2, 14511–14518 (2014).

[b19] Lu C. . Electrochemical performance and thermal property of electrospun PPESK/PVDF/PPESK composite separator for lithium-ion battery. J. Appl. Electrochem. 43, 711–720 (2013).

[b20] Kumar G. G. . Irradiated PVdF-HFP-tin oxide composite membranes for the applications of direct methanol fuel cells. J. Membr. Sci. 350, 92–100 (2010).

[b21] Inan T. Y., Dogan H. & Gungor A. PVdF-HFP membranes for fuel cell applications: effects of doping agents and coating on the membrane’s properties. Ionics 19, 629–641 (2013).

[b22] Abraham T. J. . Towards ionic liquid-based thermoelectrochemical cells for the harvesting of thermal energy. Electrochimica Acta 113, 87–93 (2013).

[b23] Abraham T. J., MacFarlane D. R. & Pringle J. M. High Seebeck coefficient redox ionic liquid electrolytes for thermal energy harvesting. Energy Environ. Sci. 6, 2639–2645 (2013).

[b24] Uhl S. . Development of flexible micro-thermo-electrochemical generators based on ionic liquids. J. Electronic Materials 43, 3758–3764 (2014).

[b25] Alzahrani H. A. H., Black J. J., Goonetilleke D., Panchompoo J. & Aldous L. Combining thermogalvanic corrosion and thermogalvanic redox couples for improved electrochemical waste heat harvesting. Electrochem. Comm. 58, 76–79 (2015).

[b26] Curcio E. & Drioli E. Membrane distillation and related operations–A review. Sep. Pur. Rev. 34, 35–86 (2005).

[b27] Yu J., Huang X., Wu C. & Jiang. P. Permittivity, thermal conductivity and thermal stability of poly(vinylidene fluoride)/graphene nanocomposites. IEEE Trans. Dielec. Elec. Insul. 18, 478–484 (2011).

[b28] Camacho L. M. . Advances in membrane distillation for water desalination and purification applications. Wat. 5, 94–196 (2013).

